# Mitral Valve Repair in the Modern Era: Insights into Techniques and Technologies with a Glimpse of the Future

**DOI:** 10.3390/jcm14207251

**Published:** 2025-10-14

**Authors:** Marco Rolando, Alessandro Affronti, Francesco Loreni, Marcello Bergonzini, Erberto Carluccio, Federico Fortuni

**Affiliations:** 1 Independent Cardiologist Researcher, 63100 Ascoli Piceno, Italy; 2 Cardiac Surgery Unit, Santa Maria Della Misericordia Hospital, 06129 Perugia, Italy; 3 Cardiology and Cardiovascular Pathophysiology, Santa Maria Della Misericordia Hospital, University of Perugia, 06129 Perugia, Italy

**Keywords:** mitral valve repair, minimally invasive cardiac surgery, robotic surgery, mitral regurgitation, transcatheter mitral valve intervention

## Abstract

Mitral valve repair has evolved significantly with the advent of advanced surgical and transcatheter techniques. Innovations such as 3D visualization, robotic surgery, and transcatheter edge-to-edge repair have improved procedural precision and expanded treatment options for high-risk patients. Emerging technologies, including transcatheter mitral valve repair, annuloplasty, and chordal systems, offer tailored solutions for complex mitral pathology. Personalized treatment strategies, guided by multimodality imaging and artificially intelligence-driven planning, are reshaping clinical decision-making. Ongoing trials and next-generation devices are poised to enhance long-term outcomes, marking a shift toward minimally invasive, precision-guided mitral valve therapy. This review aims to provide a comprehensive overview of recent technological advances, clinical applications, and future directions in mitral valve repair across surgical and interventional domains.

## 1. Introduction

Mitral regurgitation (MR) is the second most prevalent valvular heart disease requiring intervention in developed countries, affecting over three million people in the United States alone and contributing significantly to heart failure (HF) development and increased morbidity and mortality [1,2]. It is commonly classified as degenerative (DMR) or functional (FMR) in origin. DMR results from intrinsic structural abnormalities of the mitral valve (MV) apparatus, most commonly myxomatous degeneration or fibroelastic deficiency, whereas FMR arises from ventricular (v-FMR) and/or atrial (a-FMR) remodeling that distorts mitral geometry without structural MV damage [3].

Surgical mitral valve repair (SMVr) remains the gold standard for treating DMR, offering superior outcomes compared to valve replacement, particularly in terms of left ventricular (LV) function preservation and long-term durability [4,5]. In v-FMR, the benefit of SMVr remains controversial, given the high recurrence rates and uncertain long-term durability, which are also conditioned by the elevated burden of comorbidities and age-related frailty that often characterize this patient population. As a result, the management has progressively evolved and transcatheter edge-to-edge repair (TEER) is now considered the evidence-based first-line therapy in appropriately selected patients [6,7,8]. The opposite is true for a-FMR, where the surgical approach shows good results with low rates of reoperation, mortality, and recurrent MR, even acknowledging the lack of comprehensive data directly comparing transcatheter mitral valve repair (TMVr) and surgery in this context [9,10,11].

Conventional open-heart surgery is associated with increased perioperative complications, especially in elderly or comorbid patients [12]. To overcome these limitations, these years have witnessed rapid innovation in surgical technology, including visualization-assisted systems and robot-assisted (ROB) techniques, which aim to reduce surgical trauma, providing successful short- and mid-term outcomes [13,14]. Simultaneously, TMVr devices—such as TEER, annuloplasty devices, and neochordae systems—have expanded treatment options [15].

Moreover, the integration of advanced imaging, computational modeling, and individualized patient profiling has ushered in a new era of personalized therapy and optimized complication management [16,17,18].

This review explores the evolving landscape of MVr, emphasizing innovations in surgical and interventional approaches, as well as modern strategies for personalized treatment planning and complication management.

## 2. Materials and Methods

This narrative review was performed through a comprehensive search of PubMed, Embase, Cochrane, and clinical trial registries up to August 2025. Keywords included were “mitral valve repair”, “transcatheter mitral valve repair”, “minimally-invasive mitral valve repair”, “robot-assisted mitral valve repair”, “artificially intelligence in mitral valve repair”. Full-length, peer-reviewed articles published in English, together with relevant editorials and comment articles providing pertinent information, were considered for inclusion in the discussion.

## 3. Innovation in Surgical Technology

SMVr has undergone profound transformation over the past two decades, driven by the need to enhance procedural precision, reduce operative trauma, and improve patient outcomes and satisfaction. The increasing demand for less invasive, patient-tailored approaches has catalyzed the development of advanced surgical technologies. Innovations such as minimally invasive access, visualization-assisted and ROB surgery, and three-dimensional (3D) imaging, especially echocardiography, have significantly refined surgical planning and execution, showing great potential in improving surgical outcomes [19,20]. These technologies aim to preserve the durability and efficacy of traditional repair while expanding eligibility to higher-risk or anatomically complex patients. In this section, we outline the key advancements that are reshaping the surgical landscape of MVr.

### 3.1. Visualization-Assisted and 3D Reconstruction Technologies

Minimally invasive mitral valve repair (MIMVr) encompasses surgical techniques that avoid full median sternotomy. These approaches may differ in the site of access, the modality of visualization (direct vision or video-assisted techniques), and the modality of instrument manipulation, ranging from conventional hand-held long-shafted devices to fully ROB systems.

Compared to a direct-vision approach, the fully video-assisted technique, particularly when using 3D technology, may be associated with prolonged operative and cross-clamp times, yet offers enhanced visualization, maintains comparable perioperative safety, and demonstrates equivalent early clinical outcomes, with a tendency toward reduced intensive care unit length of stay [21]. Indeed, 3D video-assisted technology improves depth perception, enhances the precision of repair procedures, particularly with artificial chordae implantation, and is linked to shorter surgical times compared to conventional 2D video-assisted systems [22].

Advanced visualization platforms have also incorporated augmented reality and virtual reality interfaces, enabling immersive, surgeon- and cardiovascular imager-specific training and preprocedural planning [23,24,25]. Moreover, 3D printing of patient-specific MV models, derived from computed tomography (CT) or 3D echocardiographic datasets, enables hands-on simulation and preprocedural planning for both surgical and transcatheter repair [26,27,28]. When combined with machine learning-assisted segmentation, this approach is particularly beneficial for educational purposes, the evaluation of valve and vascular function, and procedural planning in cases of complex valve anatomy or in redo procedures with significant anatomical distortion [29,30].

### 3.2. Robot-Assisted MV Repair

ROB-MVr represents one of the most advanced developments in minimally invasive technologies, offering enhanced dexterity and superior visualization, compared to conventional open or thoracoscopic approaches. The most widely used platform, the da Vinci Surgical System, enables surgeons to perform complex MVr through small intercostal ports, using articulated instruments and high-definition 3D cameras.

Its technical complexity and longer operative times are offset by reduced invasiveness and shorter hospitalization, ensuring the same procedural success and long-term outcomes as other approaches, particularly in experienced centers [31].

In comparative analyses, ROB surgery is associated with shorter hospital stays, reduced blood loss, faster return to normal activity, and improved cosmetic outcomes compared to conventional median sternotomy [32]. Importantly, these benefits do not appear to compromise long-term results, with several centers reporting equivalent or superior repair durability and freedom from reoperation over 5–10 years of follow-up [14].

However, ROB-MVr also presents challenges, including a significant learning curve, requiring almost 200 cases to obtain the greatest reduction in operative time, and high initial costs related to equipment, training, and instrument maintenance [33]. Moreover, this technique is associated with longer aortic cross-clamp times, as well as procedural duration when compared with other minimally invasive non-ROB approaches [34]. Nevertheless, prior expertise in mini-thoracotomy MVr lends a positive effect on the initial learning curve [35].

Consequently, its adoption remains largely concentrated in high-volume centers with specialized expertise. Future developments may focus on improving accessibility and reducing costs while also exploring whether integrating robotic systems with real-time imaging and artificial intelligence (AI)-based procedural guidance could help mitigate the current limitations of this technique.

### 3.3. Minimally Invasive Surgical Approaches and Outcomes

**Figure 1:**
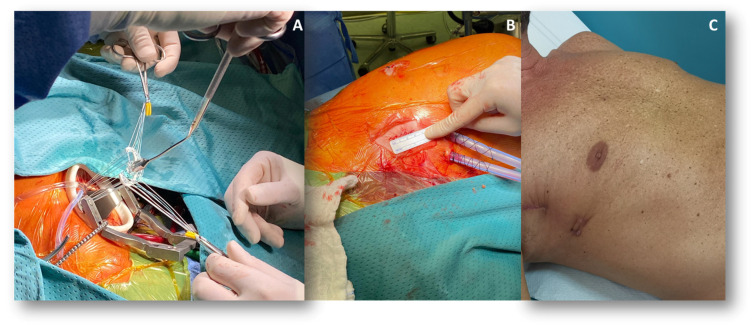
Minimally Invasive Mitral Valve Repair via Right Mini-Thoracotomy. (**A**) shows the positioning of a mitral annuloplasty band through right mini-thoracotomy access. (**B**) depicts the final result of the repair with drains in place and the incision sutured following removal of the retractor and surgical instruments. (**C**) illustrates the final surgical scar, demonstrating a superior cosmetic outcome compared with median sternotomy and reduced impact for the patient.

MIMVr has emerged as a valuable alternative to conventional sternotomy, aiming to reduce surgical trauma while preserving the high efficacy of sMVr (Figure 1). Typically performed via right mini-thoracotomy and peripheral cannulation, it offers a direct path to the MV through a 4–6 cm incision without splitting the sternum [36]. This approach is compatible with both traditional hand-held instrumentation and robotic assistance.

Multiple studies have demonstrated that MIMVr is associated with reduced postoperative pain, lower rates of postoperative complications, shorter hospital stays, improved cosmetic outcomes and faster return to daily activities, ultimately contributing to a reduction in overall healthcare costs [37,38,39]. Importantly, these benefits are not achieved at the expense of repair quality. In experienced hands, this technique has been shown to yield comparable or superior repair rates compared to the conventional approach, low mortality, and excellent long-term durability [40,41,42]. Notably, there were no differences regarding perioperative and long-term morbidity and mortality among the different sites of access [43].

**Figure 2:**
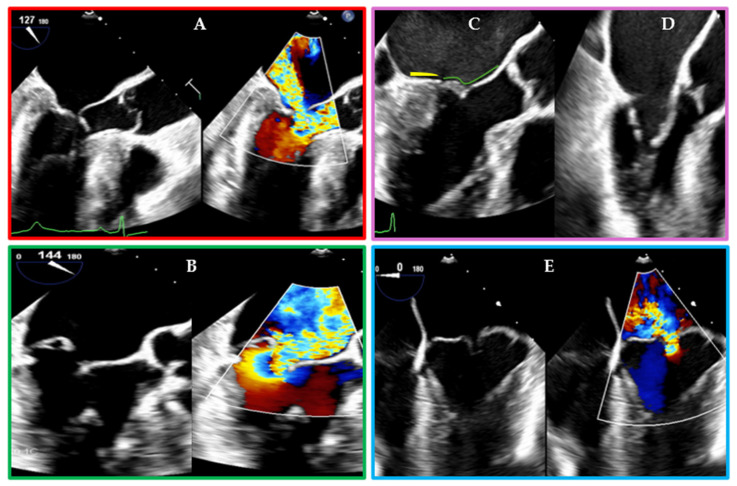
TEE Representation of Different MV Pathologies Leading to MR. (**A**) MR due to the systolic anterior motion of the MV leaflets in the presence of basal interventricular septum hypertrophy and a small LV cavity. (**B**) MR due to a flail of the posterior mitral leaflet (PML). (**C**) A case of atrial MR: note that the insertion of the PML (yellow line) is displaced posteriorly above the LV crest, making it appear short and also shifting the coaptation zone posteriorly; the green line delineates the anterior mitral leaflet. (**D**) A case of rheumatic MV disease as a cause of MR: note the diastolic doming of MV leaflets. (**E**) MR due to a bileaflet prolapse of both the anterior and the PML. In (**A**,**B**,**E**) the images are displayed with simultaneous B-mode and 2D Color.

However, patient selection remains critical. MIMVr may not be suitable in cases of complex reoperations, severe chest wall deformities, or extensive annular calcification. The technique also has a learning curve and requires the involvement of a cardiac anesthesiologist with skills in intraoperative transesophageal echocardiography (TEE), not only to specifically identify the MV pathology (as shown in Figure 2) and guide the procedure, but also to ensure correct cannula positioning during peripheral cannulation [39,44].

## 4. Progress in Interventional Therapy

The past decade has witnessed remarkable advances in TMVr, transforming the treatment landscape for patients deemed high-risk or inoperable for conventional surgery. Unlike the aortic valve position, where transcatheter techniques are now routine, the MV poses unique anatomical and functional challenges due to its complex sub-valvular apparatus, dynamic annulus, and variable pathology. Nevertheless, the evolution of catheter-based technologies—including TEER, annuloplasty, and neochordae—has enabled the successful treatment of selected MR cases through percutaneous or transapical approaches [45].

Initially targeted at patients with FMR and advanced HF, interventional techniques are increasingly being adopted in DMR, either as a bridge to surgery or as definitive therapy in frail individuals [46,47]. An ongoing randomized controlled trial is investigating the comparative efficacy and safety of TEER versus SMVr in patients aged 65 or older with DMR and no other requirements for cardiac surgery (NCT05051033).

In the following subsections, we provide an overview of the current state and future directions of TMVr therapies, highlighting their indications, technical principles, and clinical outcomes.

### 4.1. Transcatheter Edge-to-Edge Repair

TEER has become the most established form of TMVr, primarily through the use of the MitraClip (Abbott Vascular, Santa Clara, CA, USA) and, subsequently, the PASCAL system (Edwards Lifesciences, Irvine, CA, USA). Inspired by the Alfieri’s surgical technique, these devices aim to reduce MR by approximating the anterior and posterior leaflets at the site of regurgitant jet origin, thereby improving coaptation [48].

The MitraClip system has gained widespread adoption and regulatory approval in both Europe and the United States for the treatment of symptomatic DMR and FMR in high-risk or inoperable patients. Robust evidence supporting its efficacy comes from the EVEREST II trial, which demonstrated similar long-term survival and functional improvements compared to surgery, with reduced procedural invasiveness, albeit with a higher rate of residual MR and reintervention in DMR [49]. More compellingly, the COAPT trial showed that MitraClip significantly reduced HF hospitalizations and improved survival in patients with FMR who remained symptomatic despite optimal medical therapy, establishing a new standard of care in this population [8]. This evidence was further refined by the RESHAPE-HF2 and MATTERHORN trials, which demonstrated, respectively, a clinical benefit in patients with a lower degree of FMR and HF severity, and non-inferiority to the surgical approach [50,51].

The PASCAL system introduces design refinements, such as a central spacer, independent leaflet grasping—also later adopted in new-generation MitraClip devices—and broader device profiles, aimed at enhancing leaflet capture and reducing tension. Data from the CLASP study suggest favorable procedural success and MR reduction, even in anatomically challenging cases [52].

Imaging guidance, particularly with 3D TEE, plays a pivotal role in patient selection, device positioning, and the assessment of post-procedural outcomes. Although TEER offers a percutaneous and well-tolerated solution for MR, there are still limitations, particularly in the case of complex or unfeasible anatomies. Moreover, another intrinsic limitation of TEER is the absence of annular stabilization through annuloplasty, which represents a cornerstone of durable MVr in surgical practice, prompting the need to develop adjunctive therapies and combined interventions [53,54].

### 4.2. Annuloplasty Devices and Chordal Replacement Systems

Beyond TEER, transcatheter annuloplasty and chordal replacement systems have emerged as complementary or alternative strategies to address specific mechanisms of MR, particularly annular dilation and chordal rupture. These devices aim to replicate surgical techniques via catheter-based approaches and are especially relevant in FMR where annular dilation predominates, or in DMR involving isolated chordal pathology, respectively [55,56].

Among indirect annuloplasty systems, the Carillon Mitral Contour System (Cardiac Dimensions, Inc., Kirkland, WA, USA), utilizes the coronary sinus as an anchor point to apply tension along the mitral annulus, thereby reducing annular dimensions. The REDUCE-FMR trial showed that Carillon significantly reduced regurgitant volume and LV volumes compared to standard medical therapy alone [57]. However, anatomical variability in the proximity of the coronary sinus to the mitral annulus can affect device efficacy and safety, particularly due to the risk of coronary artery compression [58,59].

Among direct annuloplasty systems, the Cardioband (Edwards Lifesciences, Irvine, CA, USA) is anchored directly onto the posterior mitral annulus via a trans-septal approach and allow for on-table cinching. In a single-arm prospective multicenter study of FMR treated with the Cardioband system, the device demonstrated satisfactory safety and effectiveness. After 1 year, most patients had moderate or less MR and experienced significant improvements in functional status [60].

**Figure 3:**
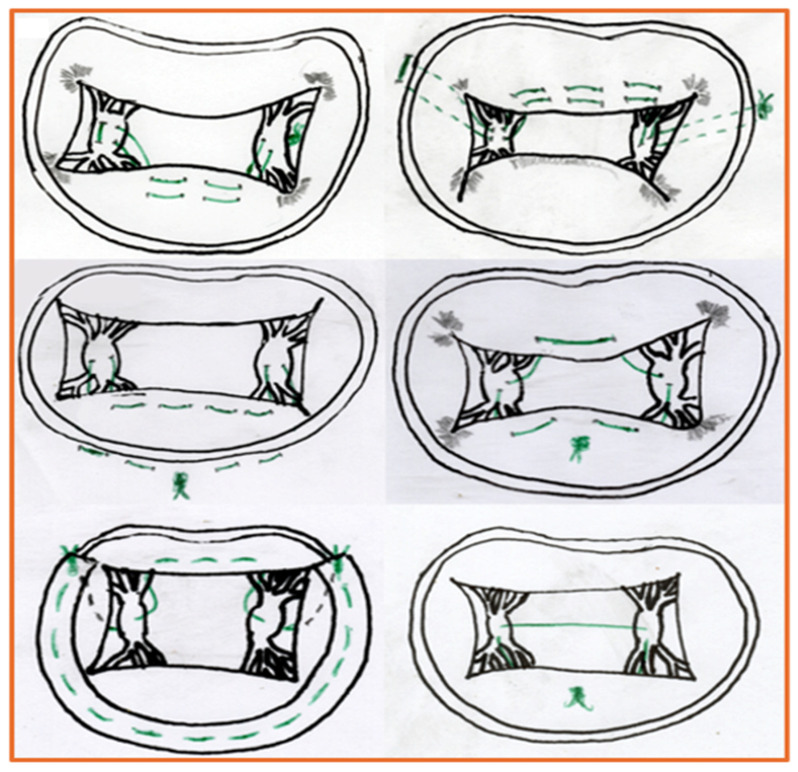
Schematic Illustration of Some Different Techniques of Surgical Cordal Implantation. From the top left, moving clockwise, the following surgical techniques are represented: Roman Arch, Minotaurus, “V” technique, Square, ARGO, and Corset. The sutures are represented in green. In some cases a dashed line was traced to better delineate the route of the suture.

In the realm of chordal replacement, the NeoChord DS1000 (NeoChord, Inc., Maple Grove, MN, USA) and HARPOON TSD-5 (Edwards Lifesciences, formerly Harpoon Medical, Inc., Baltimore, MD, USA) systems offer transapical, beating-heart access to the MV, enabling targeted implantation of artificial chords in patients with isolated leaflet prolapse. These systems have shown promising results in terms of procedural success, leaflet stabilization, and durable MR reduction in selected patients with favorable anatomy [61]. Although remaining underutilized, with limited adoption outside experienced centers, they illustrate the growing versatility of TMVr. Nevertheless, from a technical standpoint, the surgical approach allows for more complex and versatile neochord implantation compared to the transcatheter approach, where the procedure is constrained by the access site and the device capabilities (Figure 3).

Although remaining largely investigational or still in early clinical use, they illustrate the growing versatility of TMVr.

Although remaining underutilized, with limited adoption outside experienced centers they illustrate the growing versatility of TMVr.

### 4.3. Patient Selection and Procedural Outcomes

**Figure 4:**
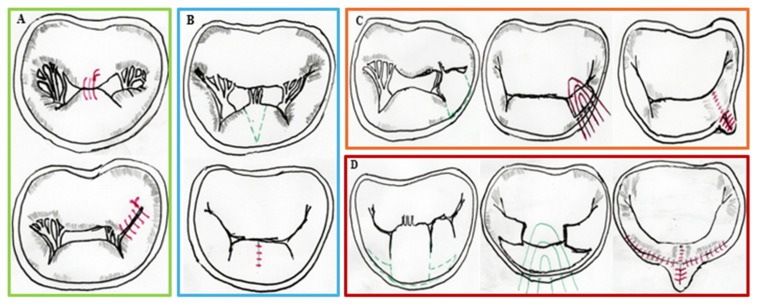
Schematic Illustrations of Principal Techniques Employed in SMVr. (**A**) Edge-to-edge suture, also known as Alfieri’s suture, in A2-P2 (**top**) and A3-P3 (**bottom**) positions. (**B**) Triangular resection. The green dashed lines indicate the site at which leaflet resection is performed (**top**). The parallel continuous red lines represent subsequent leaflet suturing following resection (**bottom**). (**C**) Quadrangular resection with annulus plication. The green dashed lines represent the site (P2M2-P3) at which resection will be performed. The continuous red lines are the annulus plication sutures that are going to be placed. The parallel continuous red lines represent the sutures at the end of the repair. (**D**) Quadrangular resection with sliding plasty. The site of resection is demarcated by the dashed green lines. The continuous green line represents the suture threads. The red lines denote the final results.

Appropriate patient selection is critical for the success of SMVr and TMVr. In surgical patients treated with a minimally invasive approach, any factor that increases the risk of complications, repair failure or conversion to full sternotomy can negate the benefits of a minimally invasive approach and may even result in worse outcomes than a planned conventional surgery. Most repairs (Figure 4) can be performed successfully, especially in DMR. However, mitral annulus calcifications or rheumatic heart disease may be challenging—if not impossible—to manage effectively with this approach.

The need for concomitant procedures—such as tricuspid valve repair, atrial fibrillation ablation, or atrial septal defect closure—is not a limitation for MIMVr and is routinely addressed through this approach in experienced centers. Nonetheless, it is important to recognize that they require additional surgical time. Similarly, previous thorax surgery performed via thoracotomy or the presence of extensive pleuropulmonary adhesions may represent a significant limitation [62]. In the presence of severe lung and pleural adhesions, dissection may be longer, and severe lung emphysema may result in prolonged ventilator dependence; these aspects must be considered. Conversely, the need for concomitant coronary artery bypass grafting represents a substantial impediment to this approach, especially when a robotic-assisted approach is planned, due to both increased complexity and prolonged operative time [63]. However, in cases with single or double vessel disease, a staged hybrid strategy involving percutaneous revascularization may offer a practical alternative. Minor aortic dilatation generally does not preclude MIMVr when a transthoracic aortic clamp is used, in contrast with ascending aortic aneurysms, also considering that endoaortic balloon occlusion is not feasible with an aortic diameter of ≥40 mm. Aortic regurgitation greater than mild may compromise the effectiveness of myocardial protection. Extensive calcification of the thoraco-abdominal aorta and iliac-femoral arteries increases the risk of cerebral embolism from retrograde flow and should be considered as a contraindication to femoral artery cannulation. In these cases, axillary artery cannulation may be a feasible alternative. Severe pulmonary hypertension is also a risk factor for post-operative complications. Among patient factors, age is not a real contraindication, but in the presence of chest wall deformities and severe obesity, or in very small patients, MIMVr is generally unfeasible [62].

Regarding the TMVr, every technique has its favorable and unfavorable anatomies [64]. In general, active endocarditis, hemodynamically significant mitral stenosis, and severe calcification in the grasping zone are clear contraindications to the procedure. If a trans-septal approach is selected, anatomies that make this procedure unfeasible must be recognized, such as interrupted inferior vena cava (e.g., caval filter) or thrombus at the interatrial septum [65,66]. In this situation, a coronary sinus annuloplasty or a transapical approach may be considered.

Procedural outcomes in MIMVr are excellent in terms of both efficacy and safety, both in the short and long term [67]. Moreover, the choice of one approach over another does not appear to negatively impact major clinical outcomes, such as short- and long-term mortality or long-term reoperation rates [68].

Regarding TMVr, differentiation between DMR and FMR must be done. In DMR, most of the published experience derives from the EVEREST II, the CLASP, the TACT, and the TRACER trials that explored the safety and efficacy of different systems of TMVr [49,69,70,71]. To date, data from the only available direct comparison study between TMVr and SMVr shows that TMVr is associated with a lower incidence of post-operative adverse events, mainly driven by a lower risk of prolonged mechanical ventilation and blood transfusion [72]. The greater risk of re-intervention is confined to the first 6 months, beyond which the difference is no longer undeniable [49]. A recent retrospective study further demonstrated that for DMR due to posterior leaflet prolapse, SMVr ensures greater durability at mid-term follow-up compared with TEER [73]. In FMR, the benefit of the transcatheter approach has been proven not only in the setting of v-FMR, but also with the newly recognized entity of a-FMR [8,74]. It is worth noting, however, that the surgical approach allows us to treat multiple pathologies in a single-stage intervention.

Another noteworthy aspect is that in both SVMr and TMVr, higher institutional monthly volumes of interventions have been associated with greater procedural success, shorter hospital stays, and improved long-term major clinical outcomes, including survival, rates of cardiac death, HF hospitalization, and re-operation [75,76].

## 5. Personalized Treatment Strategies

**Figure 5:**
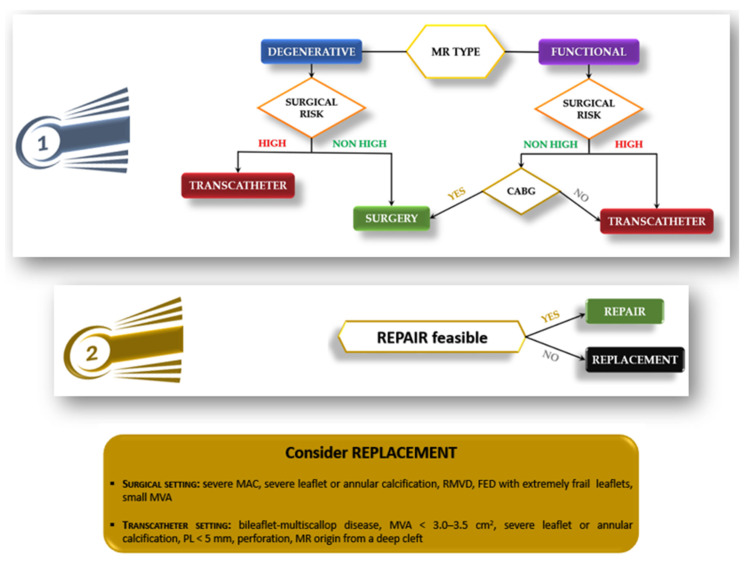
Flowchart Illustrating the Treatment Strategy for MR. (**1**) The initial step is based on the underlying etiology (DMR vs. FMR) and the patient’s surgical risk, guiding the choice between the surgical or transcatheter approach. (**2**) Once intervention is indicated, the decision between repair and replacement depends on feasibility and on specific anatomical conditions where valve replacement should be considered (highlighted in the yellow box).

As MV interventions become increasingly diverse and sophisticated, a personalized approach—guided by detailed anatomical, functional, and clinical profiling—has become essential to optimize patient outcomes [77]. Tailored treatment strategies are now central to contemporary MVr practice, particularly in high-risk and anatomically complex patients (Figure 5).

### 5.1. Integration of Imaging (TEE and CT) for Planning

Advanced cardiac imaging is central to personalized MV therapy, serving as the cornerstone for patient selection, procedural planning, and intra-procedural guidance.

A comprehensive assessment of valvular anatomy, the identification of diseased segments, and an understanding of the pathophysiological mechanism underlying MR are critical for selecting the appropriate repair strategy and achieving optimal surgical results.

**Figure 6:**
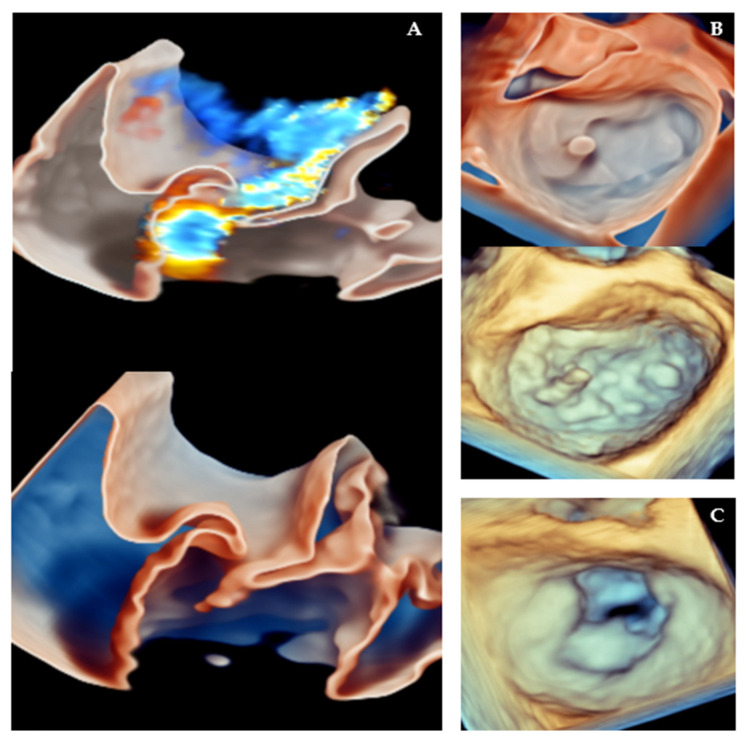
Three-dimensional TEE Representation of MV Pathologies Leading to MR. (**A**) PML flail displayed using the glass rendering modality, with (**top**) and without (**bottom**) 3D color. (**B**) “En-face” view of the MV, showing the flail of a small portion of the P1 scallop, displayed with (**top**) and without (**bottom**) the glass rendering modality. (**C**) “En-face” view of a rheumatic MV in a patient with MR (in this case MVr was not feasible).

Traditionally, 2D imaging has been the cornerstone for evaluating the MV apparatus, identifying MR mechanisms, predicting complications after MVr; however, 2D imaging has limitations in spatial orientation, depth perception, and requires mental reconstruction of the MV’s complex 3D structure, which may reduce diagnostic accuracy and confidence. Three-dimensional TEE provides a real-time en-face view of the MV (Figure 6), enhancing the surgeon’s ability to identify prolapse, flail, or cleft lesions, especially in complex bi-leaflet or commissural disease [78].

In approaches without chest cannulation, all cardio-pulmonary bypass and cardioplegia catheters are inserted percutaneously, with intraoperative 2D-TEE that guides and monitors their placement and function. The venous outflow cannulae are inserted into the inferior and superior vena cava, through the femoral and jugular vein, respectively. The arterial inflow cannula is a ballon-tipped catheter used for cardioplegia delivery, endovascular occlusion, and invasive pressure monitoring and is inserted into the descending aorta through the femoral artery. When an endovascular approach to ascending aortic cross-clamping is selected, TEE is the only modality capable of assessing proper balloon positioning and inflation, both of which are critical for effective aortic occlusion [44]. TEE also plays a pivotal role for the intraprocedural guidance of the transcatheter approach and for the post-operative evaluation of results in both SMVr and TMVr [64]. The integration of 3D imaging and reconstruction technologies—including 3D-TEE, CT, and cardiac magnetic resonance imaging—has dramatically improved preoperative and intraoperative valve assessment [44,79,80].

CT is increasingly recognized as a valuable tool for assessing anatomical feasibility and optimizing procedural planning for MIMVr. It provides precise evaluation of thoracic anatomy, aortic position, coronary artery and aortic disease, and peripheral vascular access, crucial for determining whether a minimally invasive approach is technically achievable and safe [81]. Regarding the TMVr setting, the role of CT in pre-procedural evaluation is consolidated when a direct annuloplasty and a chordal repair technique is selected. In the former, with the aim to evaluate the mitral annulus geometry and its relationship with the circumflex artery; in the latter, to identify the optimal site of access for the device [64].

### 5.2. Risk Stratification and Treatment Algorithms

The effective management of MR relies on individualized risk stratification to guide therapeutic decision-making. Traditional risk models (e.g., STS score and EuroSCORE II) provide important estimates of perioperative morbidity and mortality, but may be insufficient when used as standalone tools [82,83]. They have shown limited accuracy when applied to contemporary populations undergoing transcatheter mitral valve interventions. Furthermore, they have shown limited accuracy in the TEER population. Recognizing these limitations, dedicated models (COAPT risk score and MitraScore) have recently been developed and validated to address the specific risk profile of this patient cohort [84,85,86].

Additional assessment of frailty, comorbidity burden, functional capacity, and cognitive status has consistently been shown to improve prognostic accuracy, since these factors independently predict adverse outcomes after both surgical and transcatheter interventions [87,88]. Patient preference, often influenced by perceived quality of life and recovery expectations, also represents an essential dimension of the decision-making process.

Multidisciplinary Heart Teams integrate these parameters with anatomical and procedural considerations into treatment algorithms, ensuring that decisions reflect not only procedural feasibility but also the patient’s broader clinical context and life expectancy [7]. Shared decision-making, informed by clinical guidelines, risk assessment, and patient values, is fundamental to selecting the optimal strategy—whether SMVr, TMVr, conventional or minimally invasive approaches, or conservative management.

Emerging models are increasingly incorporating demographic, clinical, imaging-derived markers, and laboratory parameters to refine these algorithms both in surgical and transcatheter cohorts [83,84,85].

## 6. Future Directions

The landscape of MVr continues to evolve rapidly, driven by innovations in device design, imaging technology, computational modeling, and clinical research.

Future next-generation devices for transcatheter procedures should be engineered to ensure a favorable safety profile, a rapid learning curve, reduced procedural invasiveness and complexity, broader applicability across a wide range of pathologies and anatomies, and improved durability, also aiming to further enhance the personalization of care. Emerging combination therapies—integrating annuloplasty with TEER or chordal replacement—seek to replicate the comprehensiveness of surgical repair through transcatheter approaches. These strategies may be particularly beneficial in mixed pathologies, where isolated leaflet or annular strategies often prove insufficient [89,90].

Advanced imaging tools, such as 4D-CT, fusion echocardiography, and augmented reality guidance, are being developed to support precision interventions [91]. Furthermore, AI and machine learning are increasingly being integrated into procedural planning—also using virtual and augmented reality—offering predictive analytics for risk stratification, the optimization of device selection, the prediction of outcomes and complications, and the personalization of follow-up strategies [92,93].

Moreover, the continued growth of global registries and adaptive clinical trials will support iterative device development and post-market evaluation, contributing to the refinement of indications and the expansion of treatment options. In this regard, the ongoing REPAIR MR trial (NCT04198870) and PRIMARY trial (NCT05051033) will help shed further light on the field of MVr by comparing the effectiveness of surgical versus transcatheter approaches for DMR.

Taken together, these advances will drive a shift toward precision-guided, data-enhanced therapy, transforming MVr into a highly individualized, evidence-based discipline.

## 7. Conclusions

MVr has entered a new era defined by technological innovation, minimally invasive strategies, and personalized treatment pathways. Surgical advancements—such as robotic-assisted and 3D-guided techniques—have enhanced precision while reducing operative morbidity. In parallel, transcatheter therapies, particularly TEER and emerging TMVR platforms, have expanded treatment options for high-risk patients.

Ongoing clinical trials, the integration of AI-driven procedural planning, and next-generation devices are expected to further improve outcomes and durability. Central to future success will be the continued refinement of patient selection algorithms and the use of multimodality imaging to tailor therapy.

As these innovations mature, MVr is poised to become increasingly personalized, data-informed, and less invasive, offering durable, life-improving treatment to a broader range of patients.

## Data Availability

No new data were created or analyzed in this study. Data sharing is not applicable to this article.

## Abbreviations

The following abbreviations are used in this manuscript:

CT	Computed Tomography
DMR	Degenerative Mitral Regurgitation
FMR	Functional Mitral Regurgitation
HF	Heart Failure
LV	Left Ventricle/Left Ventricular
MIMVr	Minimally Invasive Mitral Valve Repair
MR	Mitral Regurgitation
MV	Mitral Valve
MVr	Mitral Valve Repair
ROB	Robot-Assisted
SMVr	Surgical Mitral Valve Repair
TEE	Transesophageal Echocardiography
TEER	Transcatheter Edge-To-Edge Repair
TMVr	Transcatheter Mitral Valve repair
